# Auditive training effects from a dichotic listening app in children with dyslexia

**DOI:** 10.1002/dys.1600

**Published:** 2018-11-12

**Authors:** Turid Helland, Frøydis Morken, Josef J. Bless, Hanna V. Valderhaug, Monica Eiken, Wenche A. Helland, Janne v.K. Torkildsen

**Affiliations:** ^1^ Department of Biological and Medical Psychology University of Bergen Bergen Norway; ^2^ Section of Research and Innovation, Helse Fonna Health Authority Helse Fonna Haugesund Norway; ^3^ Department of Special Needs Education University of Oslo Oslo Norway

**Keywords:** attention, dichotic listening, dyslexia, emergent literacy, lateralisation, training

## Abstract

Dichotic listening (DL) taps information on the brain's language laterality, processing, and attention. Research has shown that DL responses in dyslexia deviate from the typical pattern. Here, effects of DL training and its correspondence to rapid naming (RAN) and digit span (DS) in typical children and children with dyslexia were assessed. Three groups of third graders participated: two training groups, control training (CT) and dyslexia training (DT), and a control group that received no training (control no training, CnT). All took part in testing pretraining and posttraining. DL measures were on laterality, response scores, and attention. The three groups showed different response patterns: minor changes in CnT, change in all measures in CT, and some changes in DT. RAN and DS scores correlated significantly with some of the DL measures, especially with the attention scores. Our findings support arguments that brain architecture for language in dyslexia is lateralised in the same way as in children without dyslexia. However, the ability to modulate attention during DL is weaker in dyslexia than in typically developing children. A training‐induced normalisation of lateralisation was observed in free recall in the dyslexia group, which suggests that DL training may be a promising intervention approach.

## 1 INTRODUCTION

The present paper focuses on training with dichotic listening (DL) in 8‐year‐old children with dyslexia and controls, using a DL mobile application (Bless et al., 2013), which to our knowledge has not been used in dyslexia intervention before. Dyslexia affects the development of literacy skills and is characterised by difficulties with skills such as phonological processing, rapid naming (RAN), working memory, and processing speed. It has a biological basis, and although it is resistant to conventional methods of literacy teaching, it can be mitigated by appropriate and specific intervention and environmental support (BDA, 2007).

When children learn to read and write, they tend to go through three literacy stages (Frith, 1986). The preliteracy stage is before they receive formal literacy training in school. The emergent literacy stage is when children learn to read and write. The literacy stage is when reading and writing have become automatised skills, and function as tools for academic learning.

## 2 THE READING NETWORK AND LITERACY SKILLS IN DYSLEXIA

The reading network of the brain changes with age, development and environmental stimuli. In the Bergen Longitudinal Dyslexia Study (http://www.uib.no/en/project/speakup), the dyslexia group diverged from the control group at both the preliteracy and the emergent literacy stages. At the brain level, left hemisphere deficits associated with visual and auditory memory processes were seen at the first two literacy stages in children who developed dyslexia (Clark et al., 2014; Morken, Helland, Hugdahl, & Specht, 2014), but this difference levelled out when the children were at the literacy stage. At the cognitive level, the same group of children showed a variety of deficits with language processing, visual and auditory memory functions, attention, and executive functions, but degree and impact of the deficits changed with age. However, early scores of RAN and digit span seemed to be reliable predictors of later reading and spelling outcomes (Helland & Morken, 2016). Other studies have found that children's early RAN skills and literacy skills appear to have a reciprocal effect (Peterson et al., 2017; Wolff, 2014). As to more demanding writing tasks, significant correlation between digit span scores and semantic errors in sentence dictations have been reported (Morken & Helland, 2013).

## 3 LANGUAGE LATERALISATION

Typically, language lateralisation develops from more diffuse hemispheric representation in infancy to left hemispheric lateralisation in adulthood (Holland et al., 2007). Even though the lateralisation observed in infants is weaker than in adults, the brain regions involved in listening to speech are very similar to those observed in adults. Functional lateralisation consolidates by age and exposure to language. In her discussion of cerebral asymmetry and language development, Bishop (2013) argues that it is a popular notion that disruption of this lateral development leads to problems with language and literacy. Individuals with normal language development may show bilateral dominance, and different language functions may relate to either the left or right hemisphere to different degrees. Still, children with impaired language functions may show slower lateralisation development compared with typical children, or this development may not take place at all. She concludes that it is still not known if this atypical development is a cause, correlate, or consequence of language development (Bishop, 2013).

Classic in the history of dyslexia research are theories of deviant lateralisation (Geschwind & Galaburda, 1985) and remediation by language stimulating to the left (Orton, 1937) or to the dominant hemisphere (Bakker & Vinke, 1985). Also, increased literacy skills have shown to improve left hemisphere brain functions in the cortical reading network (Dehaene et al., 2010). Hence, there are reasons to believe that stimulus given to the reading network in the left hemisphere could enhance functional reading in dyslexia.

## 4 DICHOTIC LISTENING

The DL paradigm was developed and described in the early 1960s and 1970s (Kimura, 1961b; Studdert‐Kennedy, Shankweiler, & Pisoni, 1972). It has been used as a non‐invasive method to study brain lateralisation of speech perception, has been applied to many clinical disorders to assess impairments within attention, working memory and executive functions, and has been validated through numerous studies using different methods (Hugdahl, 2011).

During a typical DL assessment, the subject is presented with pairs of consonant–vowel (CV) syllables (such as /ba/ and /da/) via headphones; one syllable is played to the right ear, and the other is played simultaneously to the left ear. The common DL paradigm consists of three different instructions. In the nonforced (NF) condition, the subject is asked to report freely what he or she heard the best. In the forced‐right (FR) condition, the subject is instructed to report the stimuli played to the right ear, and in the forced‐left (FL) condition, the subject is instructed to report the stimuli played to the left ear. The right ear advantage (REA) is a benchmark finding, showing a preference to report stimuli presented to the right ear (Bryden, 1988; Hugdahl, 1995; Kimura, 1961a). Since the typical pathway for language processing is contralateral, a REA in the NF condition reflects the superior processing capacity for the right ear stimulus in the left hemisphere. This has also been validated through studies using brain imaging (Dos Santos Sequeira, Specht, Moosmann, Westerhausen, & Hugdahl, 2010; Hugdahl & Helland, 2013; Tervaniemi & Hugdahl, 2003; van den Noort, Specht, Rimol, Ersland, & Hugdahl, 2008). On the other hand, a left ear advantage (LEA) reflects the superior processing capacity for the left ear stimulus in the right hemisphere, and a no ear advantage (NEA) reflects no superior processing of any ear in either hemisphere.

Whereas a REA is seen in the majority of older children and adults, a LEA is seen in about 10% of the population (Hugdahl & Asbjørnsen, 1994). Younger children are known to have a less pronounced REA, but parallel to language development, there is a gradual shift towards the left hemisphere (Hugdahl, 2003). The scores of correct responses, to both left and right ear stimuli, indicate the degree of lateralisation. The laterality index (LI) is a measure of the relative difference between the ear scores independent of correct responses. The FR and FL conditions are seen as tests of attention. These two instructions reflect different cognitive processes. In the FR condition, the instruction to report from the right ear follows the “bottom‐up” bias towards the right ear stimulus, resulting in an increase of the REA. In the FL condition, on the other hand, the instruction is opposite to the stimuli‐driven bottom‐up perception, and demands strategies for executive cognitive control (Bless et al., 2015; Hugdahl et al., 2009). The bottom‐up processes are innate, favouring the right ear, while response to the top‐down FL condition is seen first at around the age of 10 (Takio et al., 2009). Changes in the bottom‐up and top‐down capacities may also interact with the functions of the reading network in the brain (Hugdahl & Westerhausen, 2016).

The analyses of DL can be complex, as some studies indicate that handedness, age, and gender may interact with the DL scores (Hakvoort et al., 2016; Obrzut, Boliek, & Bryden, 1997a, 1997b). However, in a large scale study of subjects ranging from age 10 to older adults, it was concluded that handedness did not affect any of the findings, and no sex differences were seen in children and older adults (Hirnstein, Westerhausen, Korsnes, & Hugdahl, 2013).

## 5 DL AND DYSLEXIA

DL studies of dyslexia have yielded mixed results (seei.e., Moncrieff & Black, 2008; Obrzut et al., 1997a, 1997b). Some studies report a lack of REA in individuals with dyslexia (Helland & Asbjørnsen, 2001; Hugdahl, Helland, Faerevaag, Lyssand, & Asbjornsen, 1995; Moncrieff & Black, 2008), while other studies have reported normal REA (Heiervang et al., 2000). Yet, other studies report enhanced frequencies of NEA or LEA in dyslexic subgroups (Helland, Asbjørnsen, Hushovd, & Hugdahl, 2008; Hugdahl & Helland, 2013). A few studies have addressed the relationship between DL scores and benchmark WM measures in dyslexia. In a study of right‐handed controls and children with dyslexia, no significant correlations between an LI and phonological processing measures were found (Hakvoort et al., 2016). The findings were tentatively explained by the different levels of processing, i.e., that the phonological measures assessed processing at the lexical level, while the DL tasks were primarily sublexical tasks. Another explanation was that the DL method (using only a laterality index) might not be refined enough to relate to phonological processing.

Using the attention shift index (ASI) lambda scoring method in a control and dyslexia group aged 12–13, two main findings were reported (Helland & Asbjørnsen, 2001). First, the dyslexia group had a significantly lower lambda score compared with controls. Second, by subgrouping the dyslexia group by language comprehension scores into a subgroup with no language comprehension problems (L+) and a subgroup with language comprehension problems (L−), a bilateral asymmetry was found in the L+ subgroup, and a right hemisphere language representation in the L− subgroup. The left ear scores did not differ between the groups, but the right ear scores did. As an explanation for these differences, the authors suggested either auditory fusing or sequential problems in the L+ subgroup, and a larger degree of impaired processing in the L− subgroup.

In another study of 80 sixth grade subjects, 40 controls and 40 with identified dyslexia, there was a significant correlation between the right ear scores and scores of RAN, language comprehension, word reading, and sentence dictation. No correlation was seen between these variables and the left ear the scores. Also, the LI score correlated significantly with scores of sentence dictation. In the same study, subgroups of dyslexia were defined by responsiveness to school intervention. The responsive subgroup showed a typical DL pattern, whereas the subgroup with no or little response to intervention showed a lack of ear advantage (Helland et al., 2008). Again, such results raise the question of whether cerebral asymmetry is a cause, a correlate, or a consequence of language development. According to Dehaene, Cohen, Morais, and Kolinsky (2015), literacy transforms the reading network of the brain and is thus an illustration of how the brain reorganises to adjust to a new cultural skill (see also Bishop, 2013; Dehaene‐Lambertz, Hertz‐Pannier, & Dubois, 2006).

Studies point to a reciprocal interaction between emergent literacy and different measures of WM (Peterson et al., 2017). Meta‐analyses have shown impairments within the WM system in individuals with different learning disabilities (Peng & Fuchs, 2016a, 2016b), which has led to speculations about the effect of training WM and whether improving WM might transfer to skills such as reading and writing. Some meta‐analyses have yielded negative results as to transfer effects and maintenance effects (Melby‐Lervåg & Hulme, 2013; Melby‐Lervåg, Redick, & Hulme, 2016; Sala & Gobet, 2017). But as working memory capacity increases with age as shown by standard scores of digit span tests (Wechsler, 2003), and as literacy is found to reorganise and accommodate the brain (Dehaene et al., 2015), it should still be plausible to find specific training methods associated with or within working memory that would transfer to literacy skills.

Ongoing research points to encouraging results from multifactorial training (Blacker, Negoita, Ewen, & Courtney, 2017). In the introduction to their research topic on improving WM in learning disabilities, Lanfranchi and Caretti (2016) defined their aim to add new evidence on the direct and transfer effects of WM training in individuals with learning disabilities. They held that early training programs aiming at literacy awareness have showed lasting improvement as to working memory subscales. Other studies suggest effects by including components of executive functions training to initiate sessions of reading training (Horowitz‐Kraus & Finucane, 2016; Horowitz‐Kraus & Holland, 2015). Horowitz‐Kraus and Finucane concluded that multicomponent intervention programs with short executive functions “warm‐up” prior to literacy training seemed to be beneficial to reading.

Since the DL paradigm is most frequently used to assess language lateralisation and processing in the brain (left hemisphere), one may intuitively ask if training using a DL paradigm should strengthen the language dominant left hemisphere. DL training effects on auditory attention in healthy adults have been reported with a significant effect for top‐down training, that is, training in focusing attention to the left ear stimuli (Soveri et al., 2013; Tallus, Soveri, Hämäläinen, Tuomainen, & Laine, 2015). Training with children is especially challenging since it is hard to motivate them to engage in repetition of meaningless syllables with no feedback as to results. However, Moncrieff and colleagues succeeded in such training of typical children aged 7–13 years. They adjusted the intensity of sound to each individual child, and concluded that this type of training improved language skills in some children (Moncrieff & Wertz, 2008).

Due to a newly developed app, DL training can be done individually and independently of a tester. The app is easy to use and gives immediate feedback, which in itself may be motivating. The only requirements on the part of the user are hearing abilities within the normal range, and the skills to read two‐letter syllables (Bless, Westerhausen, Kompus, Gudmundsen, & Hugdahl, 2014). Also, a smartphone‐based data collection proved to be an effective method to gather data from a range of different populations (Bless et al., 2015).

The present study was initiated on the background of our former study of 8‐year‐old typically developing children, where a significant correlation was found between the ability to follow writing conventions during narrative writing, and a measure of selective attention in DL (Torkildsen, Morken, Helland, & Helland, 2016). Unpublished data from the same study showed a significant correlation between text reading, DL, RAN, and digit span (DS) scores for the original whole three grade school class (*N* = 41). This is shown in Table 1.

**Table 1:** Unpublished data from initial project^a^

Text reading, words/min	DL FR_Re	DL FR_Le	DL FR_LI		DL ASI	RAN	DS
Mean (SD)	*r* = 0.264	*r* = −0.337	*r* = 0.395		*r* = 0.308	*r* = −0.563	*r* = 0.505
63.45 (28.23)	*p* = 0.110	*p* = 0.039	*p* = 0.014		*p* = 0.060	*p* < 0.001	*p* = 0.001

*Note*. DL: dichotic listening; FR: forced‐right condition; RAN: rapid naming; DS: digit span; ASI: attention shift index; Re: right ear; Le: left ear.

a Torkildsen et al., 2016. Correlation between Text reading and DL, RAN, and DS (digit span sum score of forward and backward recall) for the whole class. Not all students from the whole class could participate in the training project because of other activities, appointments, or illness (*N* = 38 for DL; *N* = 41 for RAN, and DS).

In light of these results, one may speculate if DL training with an app would change the DL response pattern in children at an early literacy stage, and if this would have any consequence as to literacy skills. Specifically, this would be of interest as to children at risk of developmental dyslexia.

To our knowledge, the DL app paradigm has not been used in training of children with dyslexia. The primary aim of the present study was to assess the effects of DL training of children with dyslexia and controls on language laterality, processing, and attention. Based on previous research, one would expect a general right ear increase in scores in the bottom‐up (NF and FR) conditions after training, and a right ear decrease in the top‐down (FL) condition after training. However, there is some evidence that this effect might be weaker or even absent in children with dyslexia. A secondary aim was to assess if any effect of training could be related to dyslexia benchmarks, such as impaired RAN and working memory skills. Since earlier research has found correspondences between these factors, a relationship was expected also in the present study, however to a weaker degree in the dyslexia group than in the typical groups. If an effect is observed, it would motivate further research on transfer of DL training to literacy skills.

## 6 METHOD

### 6.1 Participants and groups

In the present study, 47 8‐year‐old children took part in the project. All subjects participated by their own and parental consent, and the study was approved by the Regional Committees for Medical and Health Research Ethics (https://helseforskning.etikkom.no/).

A group of 31 typically developing third graders from a school at the outskirts of a larger city in Norway served as a control group. They took part in a larger scale study reported in the study of Torkildsen et al. (2016). None of the children had any identified learning disabilities according to reports from teachers and parents, and all scored within norm on the Matrix Analogies Test–Short Form (Naglieri, 1985), a test measuring general nonverbal abilities. This group was randomly split in two by their teachers; a control group receiving no DL training (control no training, CnT, *n* = 16), and a control group receiving training (control training, CT, *n* = 15). It should be noted that since some pupils had appointments during school hours (i.e., dentists, sports) during the project period, they were allotted to the CnT group.

Sixteen children, also third graders, recruited from five different schools in the same geographical area as the control group, were identified with reading and writing difficulties by their respective schools and parents and received special needs education in accordance with the Norwegian Education Act (https://www.udir.no/laring-og-trivsel/tilpasset-opplaring/) by experienced speech and language therapists. In Norway, a dyslexia diagnosis is traditionally not set until the children are at least 11 years old. In line with discussions in other countries, this is due to a “wait and see” attitude, criticised by teachers and other professionals, and change in this practice towards early identification and intervention is slowly being implemented. In concordance with the evaluation of the children's speech and language therapists, this group was defined as a dyslexia group in the present study. All participants in the dyslexia group received training, hence the labelling dyslexia training (DT). Criteria of exclusion in both groups were intellectual disability, significant hearing or visual impairment and having a different first language than Norwegian.

### 6.2 Descriptives and baseline data

Baseline data on gender, handedness, and age by group is shown in Table 2. It should be noted that the control group was tested in the fall whereas the dyslexia group was tested in the spring, hence the age difference.

**Table 2:** Baseline data by groups

	Control		Dyslexia				
	CnT *n* = 16	CT *n* = 15	DT *n* = 16				Control vs. dyslexia
Descriptives					Chi^2^	*p*	Chi^2^
Gender M/F	11/5	8/7	10/6		0.785	0.675	*ns*
Hand R/L	14/2	8/7	14/2		6.650	0.036	*ns*

One‐way ANOVA	Mean (*SD*)	Mean (*SD*)	Mean (*SD*)	*df*	*F*		Cohen's d
Age	8.22 (0.32)	8.23 (0.24)	8.78 (0.26)	44	20.980	0.0001	2.097
Baseline data							
RAN	42.88 (9.89)	50.40 (17.07)	59.19 (17.95)	44	4.520	0.02	0.835
DS	10.88 (2.63)	11.27 (2.19)	9.50 (1.46)	44	2.933	0.06	1.265

*Notes*. CnT: control no training; CT: control training; DT: dyslexia training; RAN: rapid naming (seconds); DS: digit span, forward and backward; ANOVA: analysis of variance; SD: standard deviation. Further statistical analyses: Hand, significant Chi^2^ is due to the high frequency of left handers in CT. One‐way ANOVAs were followed up by LSD test. Effect of age: CnT/CT < DT, *p* = 0.004; of RAN: CnT < DT, *p* = 0.01; of DS: CT > DT, *p* = 0.02. *t* tests. RAN: Control < Dyslexia, *t* = 2.654, *p* = 0.01; DS: Control > Dyslexia, *t* = 2.654, *p* = 0.01.Correlations (Product–Moment, one variable list) Age × RAN × DS: age: *ns*; RAN × DS: *r* = −0.367, *p* = 0.01.

Baseline tests were RAN and DS. RAN was assessed by the colour/word naming subtask of the Stroop test (Hugdahl, n.d.; Stroop, 1935). This RAN version was chosen since other versions using letters or digits may interfere with the impaired reading abilities in dyslexia. The subjects were asked to name the colours of 48 dots of six different colours (yellow, red, black, green white, and blue) as quickly as possible, and the score is the number of seconds used. In addition, we used subtests from the WISC‐III measuring forward and backward digit span (Wechsler, 2003). As instructed, the digits were presented at 1‐s intervals. The collapsed sum of the raw scores of forward and backward recall was used, since preliminary analyses showed that separate analyses made no difference to relevant results.

### 6.3 Dichotic listening

A new mobile device (MD) version of the DLCV paradigm (iDichotic) using iPod touch devices with headphones and touch screens was used for assessment pretraining and posttraining, and for training (Bless et al., 2013). The app contains a simple hearing screening. Using volume scroll bar, a 1,000 Hz tone had to be adjusted until it became “just inaudible” assessed separately for left and right ear. Via a pop‐up window, the participants were reminded to wear the left‐channel headphone on the left ear and the right‐channel headphone on the right ear, and to adjust the main volume to a comfortable level. The subjects used earphones to listen to 36 stimuli combinations of CV‐syllables/ba/, /da/, /ga/, /pa/, /ta/, /ka/, including six homonym pairs, presented simultaneously to both ears, one syllable played to the right ear and the other played to left ear. The MD version includes the three conditions from the common Bergen DL paradigm (Hugdahl, 2003): NF, FR, and FL. The NF condition is always presented first, whereas the FR and FL conditions are randomised.

The standard interstimulus interval was set to 4 ms. The syllables were shown on the touch screen, and the subjects were instructed to immediately tick the correct syllable in accordance with the given instructions. The participants had 4 s to respond before the next syllable pair was presented, and a response was counted as correct when it matched the syllable presented to either the right or left ear on each trial. If a response did not match either syllable, or no response was given, it was counted as an error, which was calculated as follows: Err = 30 − (RE þ LE correct; Bless et al., 2015). The raw scores calculation was automatic (max score 30 points) for each of the three conditions. The homonyms were not included in this scoring.

### 6.4 Procedures

The participants were supervised by master students in logopedics, who were under supervision by professionals. Using the mobile phone seemed familiar to the children, and only introductory information was needed. All three groups were tested before training with the baseline tests, RAN and DS, and the experimental test DL (NF, FR, and FL). The pretesting of the two school groups CnT and CT took place during fall. After pretesting, the CT group trained once a day for five consecutive days using the previously described DL paradigm, whereas the CnT group received no training. Finally, DL posttesting took place for both groups early the following week. The DT children were tested individually before training, trained individually for five consecutive days, and posttested the week after. This took place in spring, hence the age difference between the groups. An overview of the testing and training procedures is given in Table 3.

**Table 3:** Overview over testing and training procedures

3. grade	CnT (fall)	CT (fall)	DT (spring)
Pretesting • RAN • Working memory • DL	Individual testing by master students under supervision of professionals administered in two separate classrooms. Earphones and presets of the app were administered by the test leaders. Each test leader was responsible for two students at a time.		Individual testing in a testing room at school. Earphones and preset of the app were administered by the test leader.
Training Note: All participants seemed to be familiar with using headsets and iPhones.	No training	Collective training in classroom five consecutive days, administered by the test leader and one teacher.	Training of max two participants at a time in the school testing room, administered by the test leader.
Posttesting • DL	Ten days after pretest under as similar conditions as possible to the pretest condition. The students were stationed in two separate classrooms. Each test leader was responsible for two students at a time. The children reported that they had enjoyed taking part in the project.		During the week after the training period under as similar conditions as possible to the pretest condition. The children reported that they had enjoyed taking part in the project.

*Note*. CnT: control no training; CT: control training; DT: dyslexia training; RAN: rapid naming; DL: dichotic listening. DL assessment tools and procedures: Numbered iPods and headsets were assigned to each participant. Relevant information on age, gender, hand preference, and first language (L1) was plotted and saved together with test scores.

### 6.5 Design and statistical analyses

Two subjects from the CnT did not take part in the DL pretesting, and three other subjects did not take part in the DL posttesting. Preliminary analyses eliminating all data from these participants and using group means as substitutes yielded no main differences as to outcome. Therefore means were used to substitute missing data.

Frequencies of ear advantage (REA, LEA, and NEA) pretraining and posttraining were assessed by Chi^2^ test. A repeated measures ANOVA was used to assess group differences with the design group (CnT, CT, DT) by tasks (response scores) by repeated measure (pre, post). The task (response scores) analyses were performed on the LI, on the DL scores NF, FR, FL (using conventional percentage scores for graphical presentation and the raw scores for table presentation), and the ASI scores. LI for each group was calculated on the basis of the DL scores using the standard formula {(Re−Le)/(Re + Le)} × 100. The ASI was calculated by the formula LOG {(FR Re% × FL Le%)/(FR Le% × FL Re%)}. Also, to exploit individual changes from ASI pre to post, an ASI gain score was calculated: ASI post – ASI pre.

To assess the relationship between the baseline scores (RAN, DS) and DL scores, correlation (Product–Moment, two‐lists) was used on the whole sample and separately by groups. The DL scores were selected on the background of earlier reported findings and on preliminary all over correlations.

Due to the skewedness in the groups shown in Table 1 as to age, gender, and handedness (writing hand; Rh = right hand; Lh = left hand), preliminary analyses were performed to see if these conditions would have any effect on the main analyses. The age of the DT group was significantly higher than the control group. The only significant correlation between age and any of the variables used in the study were in NF Re% post (*r* = 0.295: *p* = 0.04), which should be explained by a significant correlation within the DT group (*r* = 0.644; *p* = 0.007) only. A one‐way ANOVA with the design gender (2: M, F) by group (3: CnT, CT, DT) showed a significant effect in DS; F(41,5) = 2.777, *p* = 0.03. LSD follow‐up test showed that this effect was due to the scores in the DT male group (8.90) being significantly lower than the female scores in the two control groups (12.40 in the CnT and 11.86 in the CT groups), *p* < 0.01. In the CT group, there was an unexpected overrepresentation of Lh (*n* = 7). Due to small numbers in the CnT and DT groups (2 Lh in each group), a one‐way ANOVA could not be executed. However, a *t* test (2‐tailed) by hand (2: Rh, Lh) executed on the CT group yielded no significant difference on 18 of the 19 tests used in the present study. A significant difference was seen on FR Re pre score: Rh score 12.25 (1.75) < Lh score 15.57 (2.88), *t*(13) = −2.742, *p* = 0.02. This difference disappeared in the one‐way ANOVA with the design by group (3: CnT, CT, DT) by hand (2: Rh, Lh) by FR Re pre. Thus, neither age, handedness, nor gender were taken into account as covariates in the main analyses. This is also in line with the earlier reported findings of (Hirnstein et al., 2013). Significant effects were set at an alpha level of *p* < 0.05. For the ANOVAs they were followed up by the LSD post hoc test. Cohen's d was used for further comparisons of mean pre‐scores and postscores within each of the subgroups.

For comparison with DL scores in other groups, unpublished data from the Bergen Longitudinal Study were provided. These are shown in Appendix 1.

## 7 RESULTS

### 7.1 Language lateralisation

As can be seen from Table 4, the frequency of REA varied for the three groups, but increased in all three groups from pretest to posttest, while the frequency of LEA and NEA decreased. The greatest change was seen in the dyslexia group, where the majority showed a LEA at pretest, which changed to a higher frequency of REA at posttest. Chi^2^ tests were conducted in the following manner: (a) within groups, REA vs. collapsed LEA/NEA pre and post; (b) between groups, REA vs. collapsed LEA/NEA pre and post in the following order: CnT vs. CT, CnT vs. DT, Ct vs. DT. However, none of these analyses reached significance by use of a Chi^2^ test.

**Table 4:** Ear advantage pretraining/posttraining

	Control				Dyslexia	
	CnT (*n* = 16)		CT (*n* = 15)		DT (*n* = 16)	
	%	n	%	n	%	n
REA pre LEA pre NEA pre	68.75	11	46.67	7	37.50	6
	25.00	4	26.67	4	56.25	9
	6.25	1	26.67	4	6.25	1
REA post LEA post NEA post	87.59	14	73.33	11	62.50	10
	6.25	1	26.67	4	31.25	5
	6.25	1	0.00	0	6.25	1

*Note*. REA: right ear advantage; LEA: left ear advantage; NEA: no ear advantage; CnT: control no training; CT: control training; DT: dyslexia training. Chi^2^ (*df* = 1): *ns* on any group comparison.

### 7.2 Laterality index

LI scores are shown in Table 5. As to the NF condition, repeated measures ANOVA showed a main effect, F (1,44) = 4.580, *p* = 0.03. LSD follow‐up test showed that this was due to a lower LI pre‐score (3.934) compared with the LI postscore (12.890). The only within group effect was in DT with a significant increase in NF LI from pretraining to posttraining for DT (*p* = 0.03). As to the FR condition, no main effect was seen, but follow‐up test showed a significant difference between CT post and DT pre and DT post (*p* = 0.05). As to the FL condition, no effects were seen.

**Table 5:** LI in the three conditions by group pretraining/posttraining

Group		Control				Dyslexia	
		CnT		CT		DT	
DL		Mean (*SD*)	Cohen's d	Mean (*SD*)	Cohen's d	Mean (*SD*)	Cohen's d
NF	LI pre LI post	7.16 (19.49)	0.512	7.22 (31.63)	0.029	−2.37 (23.75)	0.478
		16.41 (16.51)		8.55 (37.25)		13.44 (38.23)	
FR	LI pre LI post	25.51 (17.89)	0.367	20.97 (15.17)	0.316	8.36 (27.40)	0.001
		17.65 (24.49)		28.69 (31.05)		8.33 (41.74)	
FL	LI pre LI post	9.82 (15.60)	0.105	15.40 (31.85)	0.436	6.79 (30.66)	0.028
		11.72 (20.15)		1.38 (32.42)		5.81 (37.97)	

*Note*. CnT: control no training; CT: control training; DT: dyslexia training; DL: dichotic listening; NF: nonforced condition; FR: forced‐right condition; FL: forced‐left condition; LI: laterality index; SD: standard deviation.

Cohen's d showed an effect (low medium) of increased NF LI in the pretest compared with the posttest in both CnT and DT, but not in CT. In the FR LI scores, it should be noted that although at a low effect level, the CnT group showed a decrease, whereas the CT group showed an increase. As to the FL LI, CT was the only group that showed an effect (low medium) in that the pre LI score showed a Re > Le, which was not seen in the post LI score.

### 7.3 Ear scores

**Figure 1:**
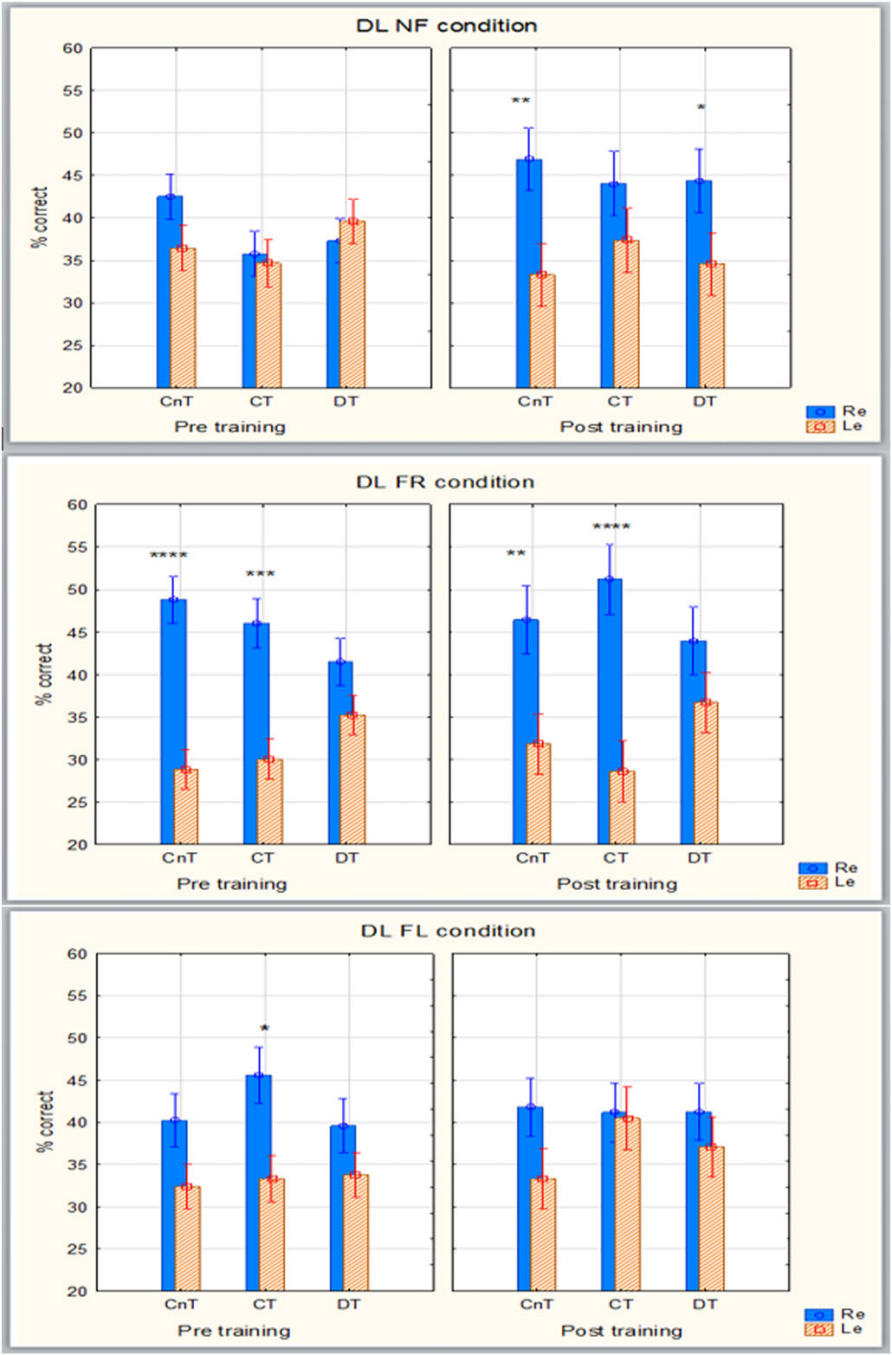
Right ear and left ear scores (%) in the three conditions by groups pre training and post training. * = *p* < 0.05; ** *p* < 0.01; *** *p* < 0.001; **** *p* < .0001 [Colour figure can be viewed at http://wileyonlinelibrary.com]

Figure 1 shows all ear scores in percentage. In the NF condition, there was a main effect of task F (1,44) = 4.147, *p* = 0.05, and an effect of interaction of repeated measures by task, F (1,44) = 6.471, *p* = 0.02. The LSD follow‐up test showed that the effect of task was due to a higher composite NF Re score (=41.86) over a composite NF Le score (=36.00), and that the interaction effect was due to the composite post NF Re scores (=45.14) being significantly higher than the composite Re pre (=38.59) and Le pre (=36.95) scores and the Le postscores (=35.05; *p* < 0.01). At the within group level, the NF Re postscores were significantly higher than the NF Le postscores in CnT (*p* = 0.001) and in DT (*p* = 0.018; see also Table 4 for raw data scores).

In the FR condition, there was a significant effect of repeated measures, F(1,44) = 24.963, *p* = 0.00001. The LSD follow‐up test showed that this effect was due to a significantly higher composite Re score (=46.25) compared with the composite Le score (=31.90). Within group analyses showed significant differences between FR Re and FR Le in the CnT and CT groups in both pretraining and posttraining (*p* < 0.001), but not in the DT group (see Table 4 for raw data scores).

In the FL condition, there was a main effect of task F(1,44) = 6.213, *p* = 0.017. The LSD follow‐up test showed that this effect was due to a significantly higher FL Re composite score (=41.56) over the composite FL Le score (=35.02; *p* = 0.016). Within group analyses showed a significant difference in CT pre (*p* = 0.03), while no significant within‐group differences were seen in the posttraining tasks (see Table 4 for raw data scores).

Raw scores and Cohen's d from the different DL tasks are shown in Table 6. The CnT column shows that the differences between Re and Le responses on the bottom‐up conditions NF and FR are mainly very large with a medium difference on the pre top‐down task. In the CT column, the bottom‐up effect changes from small in the NF condition to very large in the FR condition. In the top‐down condition, this group went from showing a large effect in the pretask to no effect in the posttask, indicating an ability to suppress stimulus in accordance with task demands. The effect in DT changed from small pretest score to medium in the NF post bottom‐up task, but with no training effect seen for the FR bottom‐up task nor for the top‐down FL task.

**Table 6:** DL raw scores showing differences between Re and Le on the DL tasks

Group	Control				Dyslexia	
	CnT		CT		DT	
	Mean (*SD*)	Cohen's d	Mean (*SD*)	Cohen's d	Mean (*SD*)	Cohen's d
DL NF						
NF Re pre	12.75 (3.15)	0.784	10.73 (3.47)	0.243	11.25 (3.17)	0.204
NF Le pre	10.94 (2.57)		10.40 (3.87)		11.88 (3.01)	
NF Re post	14.08 (3.19)	1.569	13.21 (4.99)	0.442	13.31 (4.92)	0.663
NF Le post	10.00 (2.52)		11.21 (5.06)		10.38 (5.33)	
DL FR						
FR Re pre	14.63 (3.46)	2.353	13.80 (2.83)	2.000	12.44 (3.63)	0.667
FR Le pre	8.64 (2.38)		9.00 (2.00)		10.56 (3.56)	
FR Re post	13.92 (4.52)	1.131	15.36 (4.29)	1.750	13.19 (5.55)	0.442
FR Le post	9.54 (3.34)		8.57 (4.24)		11.00 (4.98)	
DL FL						
FL Re pre	12.07 (2.79)	1.942	13.67 (4.45)	0.750	11.88 (4.05)	0.283
FL Le pre	9.71 (1.80)		10.00 (4.16)		10.13 (3.26)	
FL Re post	12.54 (3.24)	0.667	12.36 (3.99)	0.000	12.38 (4.81)	0.250
FL Le post	10.00 (3.54)		12.14 (4.42)		11.13 (4.80)	

*Note*. CnT: control no training; CT: control training; DT: dyslexia training; DL: dichotic listening; NF: nonforced condition; FR: forced‐right condition; FL: forced‐left condition; Re: right ear; Le: left ear; SD: standard deviation. Cohen's d: difference between Re and Le tasks separately for pre and post in the NF, FR, and FL conditions.

### 7.4 Attention shift index

**Figure 2:**
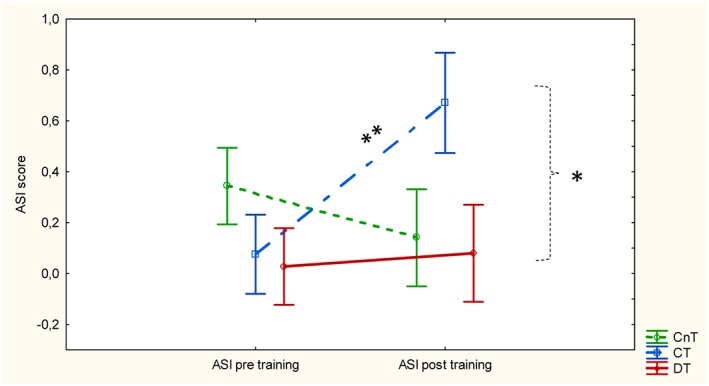
ASI pre and ASI post by groups and condition. Note: vertical bars denote +/− standard errors. * = *p* < 0.05; ** = *p* < 0.01 [Colour figure can be viewed at http://wileyonlinelibrary.com]

The ASI scores are shown in Figure 2. There were no main effects, but an interaction effect of repeated measures by group F(2,44) = 3,972, *p* = 0.03. The LSD follow‐up test showed that the ASI postscore in CT (=0.67) was significantly higher than the scores in the two other groups (CnT post: 0.14; DT pre: 0.03, post: 0.08; *p* < 0.03) except for ASI pre in CnT. The difference between CT pre (=0.08) and CT post (=0.67) was significant at *p* < 0.006, and between CT post and DT pre‐scores and postscores at *p* < 0.02, and CnT post at p = 0.03.

#### 7.4.1 ASI gain

**Figure 3:**
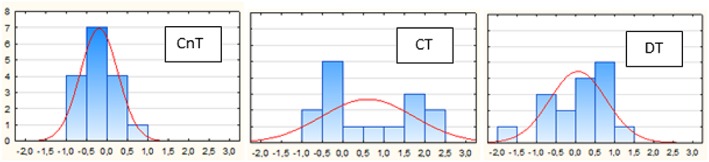
Histogram of individual changes from ASI pre‐scores to ASI postscores [Colour figure can be viewed at http://wileyonlinelibrary.com]

The histogram (Figure 3) shows individual changes in ASI scores from the pretest to the posttest. As can be seen, most individuals in the CT group improved their ASI score, whereas individuals in the CnT group showed little change. However, although not reaching significance, there were more individuals increasing their ASI scores in the DT compared with the CnT group.

### 7.5 Correlations between baseline scores (RAN, DS) and DL scores

As can be seen from Table 7, all significant correlations between the RAN and DS scores and the DL scores were moderate to strong for the entire sample.

**Table 7:** Correlations between RAN. DS and relevant DL scores

	All groups		CnT		CT		DT	
Variable	RAN	DS	RAN	DS	RAN	DS	RAN	DS
NF LI pre	−0.218 *p* = 0.141	**0.331** ***p* = 0.023**	0.250 *p* = 0.350	0.112 *p* = 0.679	−0.251 *p* = 0.367	**0.542** ***p* = 0.037**	−0.268 *p* = 0.316	0.166 *p* = 0.538
NF LI post	−0.121 *p* = 0.416	0.205 *p* = 0.167	−0.193 *p* = 0.474	0.234 *p* = 0.383	0.205 *p* = 0.464	0.249 *p* = 0.371	−0.380 *p* = 0.146	0.318 *p* = 0.230
FR LI pre	−0.202	**0.376**	−0.307	0.348	0.044	**−0.540**	0.029	0.255
	*p* = 0.174	***p* = 0.009**	*p* = 0.247	*p* = 0.187	*p* = 0.876	***p* = 0.038**	*p* = 0.915	*p* = 0.341
FR LI post	**−0.346**	**0.499**	−0.289	0.357	0.326	**−0.708**	−0.376	0.451
	**p = 0.017**	**p = 0.000**	*p* = 0.278	*p* = 0.175	*p* = 0.235	***p* = 0.003**	*p* = 0.151	*p* = 0.080
NF Re pre	−0.189 *p* = 0.202	0.101 *p* = 0.497	0.057 *p* = 0.835	0.350 *p* = 0.184	0.025 *p* = 0.930	−0.159 *p* = 0.571	−0.431 *p* = 0.096	0.067 *p* = 0.805
NF Re post	0.270 *p* = 0.067	**0.308** ***p* = 0.035**	−0.458 *p* = 0.075	0.366 *p* = 0.164	−0.004 *p* = 0.989	0.353 *p* = 0.197	−0.432 *p* = 0.095	0.320 *p* = 0.227
NF Le pre	0.165 *p* = 0.268	**−0.295** ***p* = 0.044**	−0.360 *p* = 0.171	0.176 *p* = 0.513	0.334 *p* = 0.571	**−0.655** ***p* = 0.008**	0.119 *p* = 0.661	−0.228 *p* = 0.397
NF Le post	0.165 p = 0.268	−0.147 *p* = 0.323	−0.141 *p* = 0.603	−0.056 *p* = 0.836	−0.279 *p* = 0.313	−0.194 *p* = 0.488	0.261 *p* = 0.328	−0.326 *p* = 0.219
FR Le pre	0.130	**−0.346**	0.128	−0.167	0.044	**−0.539**	−0.046	−0.301
	*p* = 0.385	***p* = 0.017**	*p* = 0.636	*p* = 0.536	p = 0.876	***p* = 0.038**	*p* = 0.867	*p* = 0.257
FR Le post	**0.293**	**−0.459**	−0.078	−0.186	0.326	**−0.708**	0.339	−0.458
	***p* = 0.046**	***p* = 0.001**	*p* = 0.775	*p* = 0.491	*p* = 0.235	***p* = 0.003**	*p* = 0.200	*p* = 0.074
ASI pre	**−0.319** ***p* = 0.029**	0.152 *p* = 0.307	−0.265 *p* = 0.321	0.097 *p* = 0.721	−0.237 *p* = 0.395	0.352 *p* = 0.198	−0.299 *p* = 0.261	−0.217 *p* = 0.420
ASI post	**−0.397** ***p* = 0.006**	**0.543** ***p* = 0.0001**	−0.384 *p* = 0.142	0.492 *p* = 0.053	−0.511 *p* = 0.051	**0.775** ***p* = 0.001**	−0.408 *p* = 0.117	0.132 *p* = 0.626

*Note*. CnT: control no training; CT: control training; DT: dyslexia training; NF: nonforced condition; FR: forced‐right condition; FL: forced‐left condition; Re: right ear; Le: left ear; RAN: rapid naming; DS: digit span; ASI: attention shift index; DL: dichotic listening.

The RAN score correlated significantly, but negatively with the FR LI score and ASI pre‐scores and ASI postscores, meaning that the fewer seconds used on the RAN task, the better was the ability to supress the Le, and to gain higher ASI scores. The significant, positive correlation with the post FR Le underlines the same tendency.

The DS correlated significantly, but negatively, with three Le scores (NF le pre, FR Le pre and FR Le post) and positively with four scores typically associated with a REA (NF LI pre, FR LI pre, FR LI post, NF Re post) and with ASI post.

When correlations were assessed for the three groups separately, correlations between DL scores and the other cognitive measures were only seen in the CT group, with no significance seen with the RAN scores (however very close to significance with ASI post), but the same correlation pattern with DS as in the All groups analyses.

Eye inspection of Appendix 1 of the different NF‐conditions reviels minor increase in scores from ages 8 to 11 compared with the increase in scores from pretest to posttest in the 8‐year‐olds in the present study.

## 8 DISCUSSION

In this study, we found that training with a DL app in two control groups and a dyslexia group yielded different response patterns. Nearly, no changes were seen in the control group with no training, major changes were seen in the trained control group, and moderate changes were seen in the dyslexia group who also received training.

### 8.1 The NF condition

In spite of variations seen in the ear advantage, laterality and magnitude scores, no significant group or ear‐score differences were seen in any of the NF pretests. The individual variations were expected since lateralisation at this age is reported to be unstable (Hugdahl, 2003; Takio et al., 2009). However, the scores of the two control groups indicate a language preference for the left hemisphere as seen in typical samples (Bless et al., 2015; Hugdahl, 2011). The lack of ear advantage in the dyslexia group is in line with what is often reported in dyslexia (Helland et al., 2008; Helland & Asbjørnsen, 2001; Hugdahl et al., 1995; Moncrieff & Black, 2008), but not in all studies (Hakvoort et al., 2016).

Interestingly, in the posttraining tests, all three groups, including the dyslexia group, changed to a higher REA frequency, to a higher LI score and to a higher right ear score over left ear score. Since this increase was seen in CnT as well as in both CT and DT, it could be attributed to a test–retest effect of two testing points with a rather short intermission. However, the DT group was the only group with significant changes on two (LI and ear scores) of the three scorings from pretest to posttest, which indicates an effect of training rather than test–retest effect. Appendix 1 with unpublished longitudinal DL data indicates a real effect of the training. The control group increased less by age from 8 to 11 than did both the CT and the DT groups in the present study from 5 days of training. Thus, the intervention led to a step towards a typical lateralisation pattern in DL with a significant right ear score over left ear score in this group. One may ask if the training promoted a development towards typical lateralisation, which would otherwise have taken place as a result of literacy training, as argued by Dehaene et al. (2015). Alternatively, it could be the case that dyslexia is not associated with atypical left hemisphere lateralisation, although the pretest scores indicated NEA for the DL group. The latter interpretation would be in line with a study of DL performance in university students with dyslexia (Kershner, 2014).

Typical traits in dyslexia in both single word reading and spelling are reversing and omitting phonemes and graphemes. The pretraining scores in DT indicate that the brain pathway to phonological processing may be relatively random, either to the left or the right hemisphere, which in return may cause confusion as to the sequencing of spoken and written language. A clinical implication of the DL training may be that it altered or stimulated the brain pathway towards a typical laterality pattern, which in turn may transfer into improved sequencing in reading and writing. Further research in emergent literacy should assess this possible outcome using non‐words and real words carefully selected and constructed to include pitfalls of reversals and omissions.

This finding also leads to further speculations regarding the earlier reported study of the responsive and nonresponsive dyslexia groups at age 12, where the responsive group showed a typical right ear advantage, whereas the nonresponsive group showed NEA (Helland et al., 2008). Seen in light of the present study the responsive dyslexia group may have had a laterality pattern resembling the present dyslexia group when they were 8 years old, but due to training the laterality pattern normalised. In contrast, the laterality pattern of the nonresponsive group may have remained atypical because they did not receive appropriate training. Conversely, having a typical lateralisation pattern from the outset may have been the reason why this group responded to training, whereas the other subgroup did not respond due to an atypical lateralisation.

### 8.2 The forced conditions

The typical response pattern of the FR task is that it synergetically follows the contralateral pathway of the brain. Hence, it is characterised as a stimulus driven bottom‐up asymmetry in language processing. The task demand causes an increased Re score over Le score and is seen in children as young as 5 years old (Takio et al., 2009). In the present study, this pattern was seen in both control groups. Also, the DT group had a higher Re than Le score, but the difference was not significant. Lack of a change to a significant REA is in line with other studies of the FR condition in dyslexia (Helland et al., 2008; Helland & Asbjørnsen, 2000; Hugdahl et al., 1995).

The posttest revealed group differences. A minor change was seen in the CnT group with a decreased, but still a significant REA, which indicates that the results in this group cannot be explained by test–retest effect, but rather by chance. The increased ear difference in the CT group in the posttest indicates an effect of training. It also underlines that the basic dominance pattern in this group is in line with what is typical (Bless et al., 2015; Hugdahl, 2011; Takio et al., 2009). In the DT group, no change was seen in the FR from the pretest to posttest scores. However, it should be noted that there was a larger, but insignificant Re score over Le score in both conditions. This could be attributed to specific difficulties with this task, which is also in line with the findings in other dyslexia samples (Hugdahl, 2015; Hugdahl & Helland, 2013; Kershner, 2014).

The FL task is seen as a stimulus driven “top‐down” task where the synergetical pathway of the brain is challenged. In the pretask, no significant difference between the ear scores were seen in the CnT and DT groups. Thus, when comparing with the FR task, the CT group showed modulation in accordance with the task demands, the CnT group showed minor modulation, and the DT groups showed no such change. Following the training, however, only the CT group switched from a significant REA in the pretest, to a NEA in the post condition. Hence, the CT group managed to suppress the Re stimuli and increase the Le response in accordance with the task demands. This shows that typically developing 8‐year‐old children are capable of cognitive control in this task if they are given training, a result which is in line with what is seen in older subjects (Bless et al., 2014). In the pretraining scores, no such cognitive control effect was seen, which is in line with the scores of the age 8–9 group as reported by Takio et al, but where larger cognitive control was seen in the age group 10–11 (Takio et al., 2009). As to the DT group, the lack of change from pretest to posttest is of special interest. If the laterality pattern in dyslexia had been dominance to the right hemisphere, as has been suggested in several studies, a LEA would have been expected in this task. This was not the case, which supports arguments that brain architecture for language in children with dyslexia is lateralised in the same way as in children without dyslexia (Bishop, 2013). But, as proposed by Kershner (2014), it is the ability to modulate attention that seems to be dysfunctional.

The ASI scores underlined the findings from the forced conditions. In contrast to the CnT and DT groups, the CT group showed a significant effect of training. However, the results of the CnT and the DT groups call for two different explanations: no change due to no training in the CnT group, and no change due to dysfunctional abilities in focusing and shifting attention in the DT group. Other studies have shown impairments within executive functions in dyslexia subgroups (Beneventi, Tønnessen, Ersland, & Hugdahl, 2010; Helland & Asbjørnsen, 2000; Smith‐Spark & Fisk, 2007), and with increasing problems especially in more demanding tasks (Reiter, Tucha, & Lange, 2005). In his study of students with dyslexia, Kershner (2014) concluded that the dysfunction relates to impaired processes in left inferior frontal gyrus, in line with what was found in 8‐year‐olds in an fMRI‐study from the Bergen Longitudinal Dyslexia Study (Morken, Helland, Hugdahl, & Specht, 2017). Another functional and structural imaging study (Hoeft et al., 2011) found that their participants with dyslexia depended on a right hemisphere pathway as a compensation for an impaired left‐hemisphere pathways in frontal regions. Likewise, Morken et al. (2014) found that 12‐year‐old children with dyslexia compensated for increasing reading demands by increased right hemisphere activation. These findings shed light on of how subjects with dyslexia implicitly handle their literacy problems seen from a brain perspective. In a clinical setting, this may explain why many students with dyslexia so easily tire when they read or write.

What implications do the above findings have for further research? As pointed out earlier, DL training effects have been seen in typical adults (Bless et al., 2014; Soveri et al., 2013; Tallus et al., 2015) and in children from 7 to 13 years old (Moncrieff & Wertz, 2008). One may speculate that these training results could be exploited further both by a longer training period and/or experimenting with sound as to intensity and frequency, as reported by Moncrieff and Wertz (2008). Moreover, DL training could be carried out in combination with literacy training. As discussed above, dyslexic adolescents showed different DL patterns (as measured by the ASI lambda score) in accordance with their language comprehension skills (Helland & Asbjørnsen, 2001). Since the ASI score showed individual training effects in both training groups, the differences between ASI pre‐scores and post‐scores should give some specific information about individual training outcomes.

As shown in Figure 3, the CnT gain scores centred around 0 (no increase), the CT scores skewed to a high frequency of increased scores. Surprisingly, at least 10 of the 16 DT subjects showed a higher ASI postscore compared with the pre‐score. Six subjects showed no increase or lower postscore. Again, one may ask if the 10 subjects showing gain compare with the earlier reported responsive dyslexia group. The six subjects with no gain, on the other hand, resemble the dyslexia subgroups where impaired language comprehension and lack of responsiveness to intervention was associated with low DL Re score (Helland et al., 2008; Helland & Asbjørnsen, 2001), respectively. In the discussion of the normalisation effect of training seen in the post NF condition, we speculated that this training might transfer to improved automatised single word reading and spelling. Since the DL tasks are also seen as tasks of perceptual and asymmetrical lateralisation, a possible effect of intensified forced condition training may be less fusing of phonemes or improved attention to phoneme sequencing. If so, more attentional capacity would be left for comprehension and fluency in reading and writing in line with the demands of simultaneous processing and storage as described by Baddeley (2003a).

### 8.3 Baseline (RAN, DS) and DL

We expected high to moderate correlations between the traditional RAN and DS scores and the DL scores at both before and after training. These expectations were only partly met. The RAN score correlated significantly with four scores, the FR LI post, the FR Le post, and the ASI pre‐scores and postscores, which points to a relationship between language processing skills and executive functions. The DS score correlated significantly with four pre‐scores, the NF LI, the FR LI, the NF Le, and the FR Le, but only FR Le reached significance at the posttests. However, the significant correlations with the NF Re post and ASI postscores indicate that the training effect interact with WM skills. Seen within the working memory model (Baddeley, 2003b), this goes for both the phonological loop and the central executive.

As Table 7 shows, the improved scores from pretest to posttest should mainly be attributed to the training effect in the CT group. There was no correlation to the RAN and DS scores in the untrained CnT group (however, the near to significant correlation between DS and ASI post is noteworthy). That this lack of relationship was also seen in the DT group, is in line with the findings of Hakvoort et al. (2016) who found no relationship between LI scores and measures of phonological processing in dyslexia, arguably because the LI measure, being primarily a measure of laterality of speech processing, was not refined enough to relate to phonological processes. The individual changes from pre to post ASI scores seen in the dyslexia group (Figure 3) indicate that the ASI score is a very sensitive measure of attentional change in language processing.

In short, this study confirmed that good language processing skills and verbal working memory skills are related to good abilities to focus and shift attention, as shown by the LI‐scores (high LI score, high performance), the Le scores (low score, high performance), and ASI scores (high scores, high performance). As described by Pennington and Bishop (2009), language disorders and dyslexia are complex, multifaceted disorders also in terms of their cognitive underpinnings. The ability to focus, switch, and sustain attention is an essential component in the coorchestration of these factors at all three literacy stages, and not least in the emergent literacy stage, when children are learning to read and write. Synthesising phonemes to be merged into real words, and keeping these words in mind to process the meaning of whole phrases or sentences, rely on these skills. In the present setting, this means that efficient RAN and immediate recall of verbally presented numbers is associated with the outcome of DL training enhancing the asymmetry of right ear responses over left ear responses. The present study indicates that language processing and sequential memory are in general associated with a trainable right ear advantage, as seen in the CT group, and to some degree in the DT group. According to our findings, the most sensitive measure of this effect is seen in the LI, Le, and ASI scores.

### 8.4 Limitations and further research

Since the emergent literacy stage forms a fundament and a platform for later literacy development, and hence academic success, it is a strategically important stage. Even more importantly, this is at an age when brain plasticity is still high, adding reasons for why specific and appropriate training methods in this period are of essential importance. Due to a limited training period and small groups, no firm conclusions should be made. However, the effects of the DL training seen in this project are promising. There is evidence of correspondence between DL, language processing, WM measures, and literacy skills. There is also evidence that benchmark brain markers and cognitive traits normalise by age and schooling, which should challenge researchers to find ways to stimulate this development to take place within the important emergent literacy stage.

As to the present study, two important findings in the dyslexia group should be pursued further. First, the switch of lateralisation pattern in the NF condition after just 5 days of training is encouraging. It could be a first step to open up for an effect of the forced conditions by more DL training, it could be quantitatively by longer training period, or qualitatively by intensified sound as described by Moncrieff and Wertz (2008). Second, the training increased the ASI scores in more than half of the children with dyslexia. Even though this increase did not reach statistical significance at the group level, there was a higher shift frequency compared with the CnT group. As other studies of dyslexia in general and more specifically of DL in dyslexia point to subgroup differences, further studies should assess this variation. There may be a responsive subgroup and a nonresponsive subgroup, which again would give new insight into the nature of dyslexia and in how to tailor training methods to individual needs.

The significance of the study is that the present findings are promising for establishing new ways of dyslexia intervention. Therefore, larger projects using the DL app in a broader setting should be encouraged. The effects both as to normalisation of brain asymmetry for language and modification of auditory perception at a vulnerable stage of literacy acquisition point to the need for longitudinal data and larger samples. As suggested above, future studies could include literacy measures and brain scanning procedures before and after DL training. Also, training with a DL app could exploit language lateralisation as a source of sound localisation. It is known that individuals with hearing impairment have problems with identifying where sounds come from (Zeitler et al., 2016). Analogous to this, one may speculate if lack of brain dominance for language may cause similar problems in literacy acquisition. A typical problem for individuals with dyslexia is the mixing and switching of phonemes and graphemes. This should be further investigated in experiments with DL training and carefully designed reading and spelling tasks as pretests and posttests. In a more practical setting, the DL paradigm should be used as executive functions “warm‐up” as suggested by Horowitz‐Kraus and Holland (2015). Training with the DL app could be given in combination with literacy training to strengthen the interaction between attention and literacy development. This study shows that children in the emergent literacy stage are both capable of and motivated for this type of training.

## APPENDIX 1

### From work in progress, unpublished DL NF data on controls and dyslexia group from the Bergen Longitudinal Dyslexia Study (BLDS) assessed with DL at the ages of 8 and 11. A comparison with CT and DT

**Table:**

		BLDS				
		Con (*n* = 28)	Dys (*n* = 13)		CT	DT
Ad Table 2. Ear advantage pre/post training						
Age 8		% (*n*)	% (*n*)	Pre	% (*n*)	% (*n*)
	REA	50.00 (14)	69.23 (9)		46.67 (7)	37.50 (6)
	LEA	35.71 (10)	23.08 (3)		26.67 (4)	56.25 (9)
	NEA	14.29 (4)	7.69 (1)		26.67 (4)	6.25 (1)
Age 11				Post		
	REA	57.14 (16)	38.46 (5)		73.33 (11)	62.50 (10)
	LEA	28.57 (8)	30.77 (4)		26.67 (4)	31.25 (5)
	NEA	14.29 (4)	30.77 (4)		0.00 (0)	6.25 (1)
Ad Table 3. Laterality index (LI) in the Nonforced condition						
Age 8		Mean (*SD*)	Mean (*SD*)		Mean (*SD*)	Mean (*SD*)
	NF LI	4.57 (26.25)	11.26 (17.93)	Pre	7.22 (31.63)	−2.37 (23.75)*
Age 11						
	NF LI	7.31 (20.86)	3.33 (15.98)	Post	8.55 (37.25)	13.44 (38.23)*
Ad Table 4. DL raw scores on NF Re and NF Le						
Age 8				Pre		
	NF Re	10.89 (3.40)	10.77 (2.65)		10.73 (3.47)	11.25 (3.17)
	NF Le	10.00 (3.49)	8.54 (2.15)		10.40 (3.87)	11.88 (3.01)
Age 11				Post		
	NF Re	12.86 (3.18)	11.93 (2.75)		13.21 (4.99)	13.31 (4.92)
	NF Le	11.06 (2.76)	11.08 (2.47)		11.21 (5.06)	10.38 (5.33)
